# The Long Non-Coding RNA FAM222A-AS1 Negatively Modulates MiR-Let-7f to Promote Colorectal Cancer Progression

**DOI:** 10.3389/fonc.2022.764621

**Published:** 2022-05-12

**Authors:** Mengmeng Song, Ye Li, Zhewen Chen, Jie Zhang, Liuqing Yang, Fan Zhang, Chunhua Song, Mingyong Miao, Wenjun Chang, Hanping Shi

**Affiliations:** ^1^Department of Gastrointestinal Surgery/Clinical Nutrition, Capital Medical University Affiliated Beijing Shijitan Hospital, Beijing, China; ^2^Beijing International Science and Technology Cooperation Base for Cancer Metabolism and Nutrition, Beijing, China; ^3^Department of Digestive Endoscopy, Shuguang Hospital, Shanghai University of Traditional Chinese Medicine, Shanghai, China; ^4^Department of Nutrition, Zhejiang Provincial People’s Hospital, Affiliated People’s Hospital, Hangzhou Medical College, Hangzhou, China; ^5^Department of Endocrinology, The Affiliated Huai’an Hospital of Xuzhou Medical University, Huai’an, China; ^6^Department of Environmental Health, Second Military Medical University, Shanghai, China; ^7^Department of Epidemiology and Statistics, Henan Key Laboratory of Tumor Epidemiology College of Public Health, Zhengzhou University, Zhengzhou, China; ^8^Department of Biochemistry, Second Military Medical University, Shanghai, China

**Keywords:** colorectal cancer, prognosis, FAM222A-AS1, MYH9, miR-let-7f

## Abstract

Accumulating evidence indicates that lncRNAs are potential biomarkers and key regulators of tumor development and progression. The present study aimed to screen abnormal expression lncRNAs and investigate the mechanisms underlying the function in the progression of colorectal cancer (CRC). Potential CRC prognosis-associated dysregulated lncRNAs were screened and identified using bioinformatics analysis. Loss/gain-of-function experiments were performed to detect the biological roles of FAM222A-AS1 in CRC cell phenotypes *in vitro* and *in vivo*. The potential microRNAs that interact with FAM222A-AS1 were identified using online tools and were verified using qRT-PCR and luciferase reporter assay. The expression of FAM222A-AS1 is significantly upregulated in CRC tumor samples and cell lines. CRC patients with elevated FAM222A-AS1 expression in the tumor samples had unfavorable overall survival and disease-free survival. Silencing FAM222A-AS1 expression significantly inhibited CRC cell proliferation, migration, and invasion both *in vitro* and *in vivo*. Furthermore, FAM222A-AS1 was mainly distributed in the cytoplasm. It may directly bound to miR-let-7f and inhibit its expression and upregulate MYH9. In summary, FAM222A-AS1, as a novel oncogene in CRC, may promote the CRC progression by inhibiting miR-let-7f/MYH9 axis. The FAM222A-AS1/miR-let-7f/MYH9 signaling pathway may be a novel valuable target for inhibiting CRC.

## Introduction

Colorectal cancer (CRC) is the second leading cause of tumor death worldwide, accounting for approximately 10% of all cases (1). An estimated 1.8 million new colorectal cancer cases and 881,000 deaths were recorded globally in 2018 (1). The incidence of CRC worldwide is predicted to increase to 2.5 million new cases in 2035 (2). However, only highly developed countries have stabilized or decreased trends in the past decades. A large number of patients initially or sequentially suffer from distant metastases, leading to a poor 5-year cancer-specific survival rate of 10-20%, which greatly hinders treatment success (3). Moreover, many CRC biomarkers have been explored, but few have been validated. Hence, it is urgent to explore and elucidate more valid prognosis-associated biomarkers of CRC.

Long noncoding RNAs (lncRNAs) are transcripts with more than 200 nucleotides that have no protein-coding capacity, and were previously regarded as transcription “noise” (4). In recent years, there has been growing evidence that lncRNAs play critical regulatory roles in diverse biological processes and the occurrence and development of cardiovascular disease (5), cardiomyopathy (6), and mesenchymal stem cells (7). Dysregulation of lncRNAs has been discovered in various human cancers (8–11). Emerging studies have suggested that lncRNAs are involved in patient outcome (12), as well as cell proliferation (13), cell migration, invasion (14), apoptosis (15) and drug resistance (16) of patients with cancer. For instance, lncRNA MALAT1 is overexpressed in colon cancer and facilitates cell proliferation by sponging miR-129-5p (17). LncRNA SNHG1 overexpression inhibits apoptosis in colon cancer, possibly *via* the Wnt/β-catenin signaling pathway (18). LncRNA ZEB1-AS1 promotes cell migration and invasion by increasing PAK2 expression and sponging miR-455-3p (12). In addition, numerous dysregulated lncRNAs are reportedly correlated with CRC prognosis. High expression of lncRNA ZEB1-AS1 was associated with metastasis and poor overall survival of CRC, similar to FAM83H-AS1, LINC01296, LINC01234, PCAT6, and PVT1 (11). However, there are still many unknown lncRNAs in CRC, and their roles require urgent investigation.

In the present study, we analyzed a bioinformatics database to screen lncRNAs that are differentially expressed in CRC and associated with the prognosis of CRC. LncRNAs FAM222A-AS1, FEZF1-AS1, and FAM83H-AS1 were significantly overexpressed in CRC tissues and associated with poor prognosis in CRC patients. Through reviewing relevant literature, we found that FAM222A-AS1 is the most novel lncRNA that has not been thoroughly studied, so this study selected FAM222A-AS1 for in-depth exploration of its functions and mechanisms. We found that FAM222A-AS1 may function as a tumor promoter by sponging miR-let-7f to protect MYH9 from degradation, promoting the growth and progression of CRC cells *in vitro* and *in vivo*. Moreover, FAM222A-AS1 overexpression could highly activate AKT1/2 and GSK-3α/β signaling, implying that FAM222A-AS1 may promote CRC *via* a more complex signaling pathway. In conclusion, FAM222A-AS1 may serve as a prognosis and therapy target for CRC.

## Materials and Methods

### Bioinformatic Analysis

Based on previous studies (19), a list of 861 CRC-associated dysregulated lncRNAs was obtained from The Cancer LncRNome Atlas (TCLA) (http://tcla.fcgportal.org/). The information on lncRNA names were confirmed using the GENCODE data databasehttps://www.gencodegenes.org/# FPKM RNA expression values of 861 lncRNAs were accessed from the MiTranscriptome (http://mitranscriptome.org/) database. They were collated with the patients’ ID of 268 CRC cases (196 colon cancer and 72 rectal cancer) from the 7256 RNA sequencing library obtained in previous studies (20). Finally, the CRC dysregulated lncRNAs (including upregulated and downregulated lncRNAs) with prognostic information were obtained. The optimal cutoff point values of the lncRNA expressions were calculated using R (“Maxstat” package). Kaplan-Meier curves were generated to evaluate the association of these lncRNAs with the overall survival of patients with colorectal cancer using R (“survminer” and “survival” survival’ packages). The expression of candidate lncRNAs was downloaded from UCSC Xena (https://xena.ucsc.edu/) to verify the differential expression of lncRNAs in colorectal cancer and normal tissues.

### Patients and Samples

Fifty pairs of colon cancer and matched para-carcinoma tissues were acquired at Changhai Hospital, Second Military Medical University, from colon cancer patients who underwent surgical resection between January 2015 and January 2020. Two pathologists independently identified the resected samples. Baseline information on the patients included age, sex, disease location, tumor family history, CEA, CA-199, mutation of B-raf and K-ras, postoperative therapy, tumor size, depth of invasion, and number of examined lymph nodes. None of the patients received local or systemic therapy before surgery. This study conformed to the standards set by the Declaration of Helsinki and was reviewed and approved by the Ethics Committee of Changhai Hospital. Written informed consent was obtained from all participants.

### Cell Lines

Human CRC cells (SW620, RKO, HT29, SW480, HCT116, and Caco2), a normal colorectal cell line (FHC), and HEK293T cells were purchased from the Cell Bank of the Chinese Academy of Sciences (Shanghai, China). The above cell lines were authenticated using short tandem repeat analysis by the Genetic Testing Biotechnology Corporation (Suzhou, China). The RKO, HCT116, HEK293T, SW620, and HT29 cells were maintained in RPMI-1640 medium (Gibco, US), and SW480 and Caco2 cells were maintained in DMEM (Gibco, US) supplemented with 10% fetal bovine serum (FBS) (Gibco, US) and 1% penicillin/streptomycin (Gibco, US). All cells were cultured at 37°C in a humidified incubator with 5% CO_2_.

### Total RNA Isolation and Quantitative Real-Time Polymerase Chain Reaction (qRT-PCR)

Total RNA was extracted from human CRC tissues or cultured cell lines using RNAiso Plus reagent (Takara, Shiga, Japan) according to the manufacturer’s instructions. RNA concentration and purity were evaluated using a Nanodrop 2000 (Thermo Scientific, Wilmington, DE, USA). An aliquot of 2 μg total RNA was reverse-transcribed into complementary DNA (cDNA) according to the manufacturer’s protocol using a PrimeScript RT reagent kit (Takara, Shiga, Japan). The relative expression levels of lncRNAs were examined by qRT-PCR using TB Green Premix Ex Taq™ II (Tli RNaseH Plus) (Takara, Shiga, Japan) in a LightCycler 480II system (Roche, Basel, Switzerland). The candidate lncRNA expression levels were calculated using the 2^−ΔΔCt^ method, which was normalized to β-actin mRNA. All assays were performed in triplicate. The details of the qRT-PCR primers used in this study are listed in **Table S1**.

### siRNA Design Transient Transfection

Two pairs of siRNAs targeting FAM222A-AS1 were designed on an online website (http://sirna.wi.mit.edu/home.php) and synthesized at Shanghai Genepharma (Shanghai, China). The siRNA-FAM222A-AS1 and siRNA control sequences were as follows: siRNA1, 5’-CAGCUUCAGUACUACGAUGUU-3’; siRNA2, 5’-UCUGAGAAUCGAUACUGAAUU-3’; siRNA-control, 5’-UUCUCCGAACGUGUCACGUTT-3’. Transient transfection of small interfering RNAs was performed using the Lipofectamine RNAiMAX reagent (Invitrogen, Carlsbad, CA, USA) at a final concentration of 20nM, according to the manufacturer’s instructions.

### Construction of Stable Cell Lines

To construct FAM222A-AS1 stably silenced colon cancer cells, the shRNA targeting FAM222A-AS1 was generated and synthesized as previously described (21) based on the sequence of siRNA1. The templates were subjected to PCR amplification with primers containing XhoI or EcoRI restriction sites (the details of the primers are listed in **Table S1**). The PCR products were purified and cloned into the pINDUCER10 vector (22). Lentiviruses were packaged into HEK293T cells using the Lenti-X HTX Packaging System (Clontech Laboratories, Inc., CA, USA) to produce lentiviral particles. SW620 and HCT116 cells were infected with FAM222A-AS1 shRNA lentivirus. Ninety-six hours after infection, the cells were treated with puromycin (2.5 μg/ml) for four weeks to select FAM222A-AS1 depleted cells. The shFAM222A-AS1 sequence used was: AGCCACAGATGTAAAGTTAACGTATGGCGAGATCTGCCTACTGCCTCGGA.

To construct colon cancer cells with stably overexpressed FAM222A-AS1, the total length cDNA sequence of FAM222A-AS1 (NCBI reference sequence NR_026661.2 for variant 1) was synthesized and commercially confirmed (Sangon Biotech, Shanghai), and was subcloned into the pENTR3C entry vector. Thereafter, sequences of FAM222A-AS1 in pENTER3C were Gateway-recombined into the pINDUCER20 vector (22). All recombinant vectors were re-sequenced by Sangon Biotech (Shanghai, China). FAM222A-AS1 overexpression plasmid pINDUCER20-FAM222A-AS1 was transfected into HT29 and Caco2 cells. Forty-eight hours after transfection, HT29 and Caco2 cells were treated with G418 (1000 μg/ml) for four weeks to select FAM222A-AS1 overexpressed cells. FAM222A-AS1 depleted cells and FAM222A-AS1 overexpressed cells were induced using 2.5 μg/mL doxycycline (Dox). The overexpression and depletion efficiencies were verified using qRT-PCR, as described above.

### Cell Proliferation, Migration, and Invasion Assays

A Cell Counting Kit 8 (CCK8) assay (Dojindo, Kumamoto, Japan) was used to measure cell proliferation in 96-well plates. Cells were seeded in triplicate at 2500 cells per well. CCK8 reagent was added at 0, 24, 48, and 72 h after transfecting FAM222A-AS1 siRNA and control siRNA, and incubated at 37°C for 2 h. Cell numbers were determined by measuring the absorbance at 450 nm using the Multiskan FC 96-well plate reader (ThermoFisher, USA). In the transwell invasion assay, 1 × 10^5^ CRC cells were seeded into the upper chamber of an insert (24‐well plates, 8 μm pore size; Corning, NY, USA) coated with Matrigel (BD Biocoat, Corning) in serum-free media, which is unnecessary for the migration assay. Media containing 20% FBS was added to the lower chamber. After culturing for 24 h at 37°C, the cells remaining on the upper membrane were removed with cotton swabs, and migrated or invaded cells were fixed and stained with 0.1% crystal violet and counted in three random high magnification fields by microscopic observation. About 2 × 10^5^cells were seeded in 12-well plates to do the wound healing assay. Draw three parallel lines on the bottom of the 12-well plates before seeding cells, and three parallel lines perpendicular to it in order to accurately locate the cell imaging area when taking pictures later. A straight scratch was made using a 10 μl pipette tip before imaging. The scratch areas were photographed again after 24h, 48h, 72h, and analyzed. For the colony formation assay, CRC cells (500 cells/well) transfected with the indicated vector were plated in 6 cm culture dish and incubated at 37°C. The cell medium was replaced every three days. Two weeks later, the cells were fixed and stained with 0.1% crystal violet. The number of visible colonies was counted using the ImageJ software.

### Animal Studies

Male BALB/c nude mice (4-5 weeks old) were purchased from the SLAC Laboratory Animal Co. (Shanghai, China). All mice were housed in laminar flow cabinets under specific pathogen-free conditions at room temperature with a 12 h light/dark cycle, with food and water available ad libitum. To establish human tumors in nude mice, SW620 and HCT116 cells (1 × 10^7^ cells) with or without FAM222A-AS1 knock down and HT29 cells with or without FAM222A-AS1 overexpression were suspended in 200 μl PBS and subcutaneously injected into the right back flank of mice. The mice were then divided into treatment (+Dox) and control groups (-Dox) (five mice per group). The mice were fed water with 2 mg/mL Dox and 5% sucrose (+Dox) or standard water and 5% sucrose (-Dox). The tumor volume was measured every three days using external measurements. The mice were sacrificed after 27 days, and the tumor volume was calculated by a formula {1/2[length(mm) × width(mm)^2^]}. The efficiency of FAM222A-AS1 knockdown was examined by qRT-PCR using tissue lysates. All procedures were performed in accordance with the National Research Council’s Guide for the Care and Use of Laboratory Animals.

### RNA Fluorescence *In Situ* Hybridization (FISH)

Cell smears were prepared for FISH using standard methods. HCT116 cells were washed twice with phosphate-buffered saline (PBS) and fixed in 4% paraformaldehyde at room temperature for 15 min. The pre-hybrid solution was added to the slides and incubated at 37°C for 1 h. The slides were incubated overnight at 60°C in a hybridization solution with the probe, washed, and dehydrated. After staining with DAPI, the slices were scanned and imaged using a fluorescence microscope (Nikon, Japan). FAM222A-AS1 emits green light, and the cell nucleus emits blue light. All experiments were repeated three times. All FISH probes were commercially synthesized by Zeheng Co., Ltd. The sequence probe used was 5’-AGTGTCATCTGGTGGCATCCCTCCTTCCCTCTTTTGGTCTCCATTTCCAT-3’.

### RNA Pull-Down for miRNA

The RNA pulldown assay was performed using an RNA pulldown kit. All subsequent experiments were performed following the manufacturer’s instructions. The sequence of the biotin-coupled FAM222A-AS1 probe was AACTTCTGAACTTGCCATCC. The sequence of the negative control probe was TTCTCCGAACGTGTCACGT. Cells were crosslinked with formaldehyde, equilibrated in glycine buffer, and scraped with lysis buffer. The cell samples were sonicated and centrifuged. The supernatant was transferred to a 2 ml tube, and separately saved 50 μl as input analysis. Cell lysates were incubated with a biotin-tagged specific probe or control probe for 3 h. The supernatant lysate was incubated with streptavidin beads for 1 h with rotation. The bead-sample mixture was washed. Subsequently, 10% of the bead sample mixture was re-purified using TRIzol. The purified mRNA was detected using qRT-PCR. The bead-sample mixture (90%) was resuspended in DNase buffer, and protein was eluted with a cocktail of RNase A, RNase H, and DNase I at 37°C for 30 sec. To explore the molecular characteristics of proteins between samples, the protein eluent was analyzed using liquid mass spectrometry (LC-MS/MS). Thereafter, GO and KEGG pathway analyses were performed to describe and analyze the basic information and functions of FAM222-AS1.

### Luciferase Reporter Assay

The human genes FAM222A-AS1, and MYH9 3’-UTR sequences were synthesized. Synthesized sequences were ligated into the pmiR-GLO dual luciferase miRNA target expression vector (Promega, Madison, WI, USA) to obtain the recombinant vectors which were named pmiR-GLO-222A and pmiR-GLO-MYH9, respectively. Luciferase reporter assays were performed as described previously (23). Cells plated in a 48-well plate were co-transfected with 50 nM miRNA mimics or negative control oligonucleotides and, 50 ng of firefly luciferase reporter using the JetPRIME reagent (Polyplus-transfection). Cells were collected 36 h after the last transfection and analyzed using the Dual-Luciferase Reporter Assay System (Promega). All experiments were performed in triplicate. The sequences of miR-let-7f and control mimics were as follows: miR-let-7f mimics, 5’-UGAGGUAGUAGAUUGUAUAGUU-3’ and 5’-CUAUACAAUCUACCUCAUU-3’; negative control mimics, 5’-UUCUCCGAACGUGUCACGUT-3’ and 5’-ACGUGACACGUUCGGAGAATT-3’.

### The Human Phospho-Kinase Array

The human phospho-kinase array kit (Art.No.: ARY003B; R&D Systems; MN Minnesota) was used to identify the FAM222A-AS1-relatived phospho-kinase proteins according to the instruction. Briefly, 1.0 mL of array buffer was first added to each well for 1 h at room temperature. After washing, CRC cell lysates (400μg/well) were added to each well overnight in the array membranes at 4°C. The membranes were then washed and incubated with the detection antibodies for 2 h at room temperature. Next, the membranes were exposed to streptavidin-HRP for 30 min on a rocking platform. After washing, protein bands were detected by enhanced chemiluminescence for 1 min and exposed to the film. The experiment was performed in triplicate.

### Statistical Analysis

All statistical analyses were performed using SPSS version 21.0 software (SPSS, IBM, Armonk, NY, USA), R (R, Version 4.0.1), and GraphPad Prism 7 software (La Jolla, USA). Data represent the mean ± standard deviation (SD). The significance of the differences between the groups was evaluated using a paired two-tailed Student’s t-test or χ2 test. The optimal cut-off values of the relative expression of candidate lncRNAs in CRC were determined by R software using “maxstat,” “survival”, and “survminer” packages. The Kaplan-Meier method was used to evaluate overall survival (OS), and the differences were compared using the log-rank test. The Cox proportional hazards model was used to determine independent factors, which were based on the variables selected by univariate analysis. Differences were considered statistically significant at p < 0.05.

## Results

### FAM222A-AS1 Is Overexpressed in CRC Tissues and Significantly Correlated With Poor Prognosis

**Figure 1:**
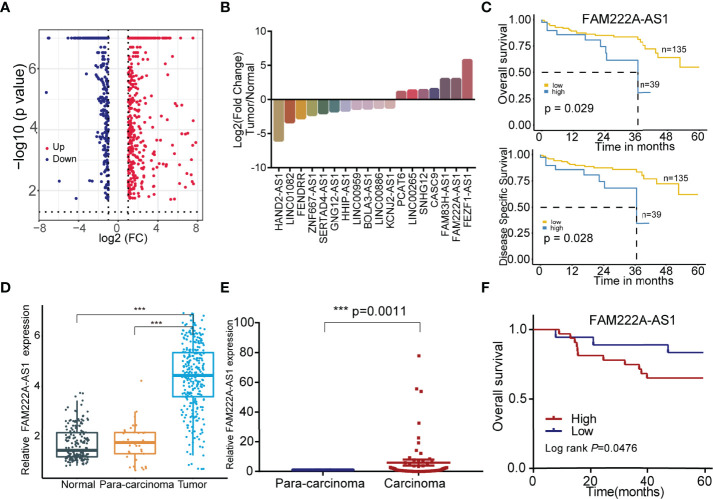
The screening of the CRC prognosis-relative dysregulated lncRNAs. **(A)** The colorectal cancer-related dysregulation lncRNAs based on the bioinformatic screening. **(B)** 18 colorectal cancer prognosis-associated lncRNAs, including 11 downregulation and 7 upregulation. **(C)** The Kaplan-Meier curves of FAM222A-AS1 based on the TCGA database. **(D)** The expression of FAM222A-AS1 in the colorectal normal tissues, para-carcinoma tissues and tumor tissues from the TCGA database. **(E)** The expression of FAM222A-AS1 in the 50 pairs clinical colorectal para-carcinoma and cancer tissues. **(F)** Kaplan-Meier analysis showed the association between FAM222A-AS1 expression and overall survival of CRC patients (n = 50).FC, Fold change. ***P < 0.001.

To explore the CRC prognosis-associated lncRNAs, we utilized bioinformatics analysis and discovered that 18 candidate lncRNAs were differentially expressed between normal colorectal cells and CRC cells, among which 11 lncRNAs were downregulated and 7 lncRNAs were upregulated (**Figures 1A, B**). The details of these lncRNAs are shown in **Tables S2, S3**. In addition, compared to low FAM222A-AS1 expression, high FAM222A-AS1 expression in CRC patients was significantly associated with a lower overall survival rate and a lower disease-free survival rate (**Figure 1C**). FAM222A-AS1, FAM83H-AS1, and FEZF1-AS1 were the most upregulated lncRNAs among the 18 candidates (**Figure 1D**). According the previous study, FAM222A-AS1 was the only unexplored lncRNA in CRC, suggesting that FAM222A-AS1 may be a potential target of CRC.

To validate the bioinformatics results, we assessed the expression of FAM222A-AS1 in 50 CRC samples and pair-matched normal samples using qRT-PCR. FAM222A-AS1 expression in CRC tissues was higher than that in para-carcinoma tissues (**Figure 1E**) and higher FAM222A-AS1 expression was associated with poor overall survival of CRC patients (**Figure 1F**). The results of GEPIA also showed that the expression of FAM222A-AS1 in tumor tissues was higher than that in normal tissues (**Figure S1**). However, there were no significant differences in the characteristics of CRC patients with high and low FAM222A-AS1 levels (**Table S4**).

Further sequence analysis showed that FAM222A-AS1 was 868 nt long and contained four exons. The secondary structure and minimum free energy of FAM222A-AS1 according to the RNAfold database (http://rna.tbi.univie.ac.at/cgi-bin/RNAWebSuite/RNAfold.cgi) are shown in **Figure S2**.

### FAM222A-AS1 Promote the Proliferation, Migration, and Invasion of CRC Cells

**Figure 2:**
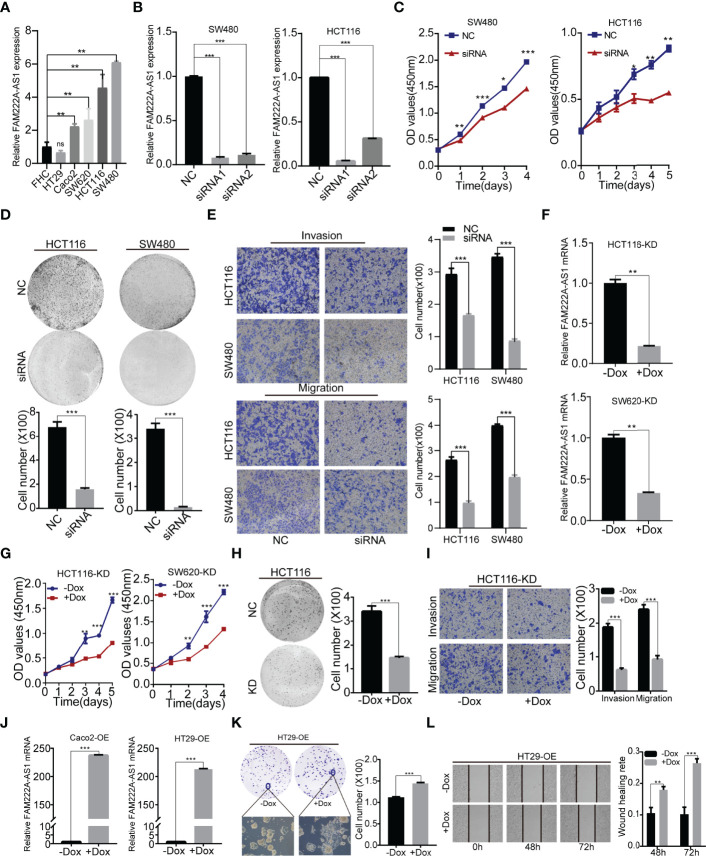
FAM222A-AS1 knockdown inhibited CRC cell proliferation, migration and invasion *in vitro*. **(A)** The expression of FAM222A-AS1 in normal colorectal cell (FHC) and six CRC cells (HT29, Caco2, SW620, SW480 and HCT116). **(B)** The expression of FAM222A-AS1 in SW480 and HCT116 after transfection with si-NC or si-FAM222A-AS1 were detected by RT-PCR. (C-D). The effects of FAM222A-AS1 knockdown on the proliferation of SW480 and HCT116 cells were examined by CCK-8 assay **(C)** and colony formation assays **(D)**. Experiments were performed in triplicate. **(E)** Transwell assays were used to detect the migration and invasion of SW480 and HCT116 cells after FAM222A-AS1 knockdown. Columns are the average of three independent viewing fields. **(F)** RT-PCR was used to determine the knockdown efficiency of the FAM222A-AS1-shRNA vector in HCT116 and SW620 cells after induced by Dox (2.5 ug/mL). **(G)** The effects of FAM222A-AS1 knockdown (induced by Dox) on the proliferation of SW620 and HCT116 cells were examined by CCK-8 assay. Experiments were performed in triplicate. **(H)** The effects of FAM222A-AS1 knockdown (induced by Dox) on the proliferation of HCT116 cells were examined by colony formation assays. **(I)** Transwell assays were used to detect the migration and invasion of HCT116 cells after FAM222A-AS1 knockdown induced by Dox. Columns are the average of three independent viewing fields. **(J)** RT-PCR was used to determine the overexpression efficiency of the FAM222A-AS1-overexpression vector in Caco2 and HT29 cells after induced by Dox (2.5 ug/mL). **(K)** The effects of FAM222A-AS1 overexpression (induced by Dox) on the proliferation of Caco2 and HT29 cells were examined by colony formation assay. **(L)** Scratch assays were used to detect the migration of Caco2 and HT29 cells after FAM222A-AS1 overexpression induced by Dox. Columns are the average of three independent viewing fields.NC, si-NC; siRNA, si-FAM222A-AS1. Caco2-OE, Caco2 stable cells transinfected with FAM222A-AS1 overexpression vectors. HT29-OE, HT29 stable cells transinfected with FAM222A-AS1 overexpression vectors; -Dox, the control group without Dox; +Dox, FAM222A-AS1 was knockdown or overexpressed induced by Dox (2.5μg/mL). (Ns, no significantly difference; *P<0.05, **P < 0.01 and ***P < 0.001).

Furthermore, we investigated the expression of FAM222A-AS1 in six CRC cell lines (RKO, SW480, SW620, Caco2, HCT116 and HT29) and a normal colorectal cell line (FHC). The results showed that FAM222A-AS1 expression in the RKO, SW480, SW620, Caco2, and HCT116 cell lines was higher than that in FHC and HT29 cells (**Figure 2A**).

To identify the biological function of FAM222A-AS1, we designed and synthesized siRNAs (siRNA1 and siRNA2) specifically targeting FAM222A-AS1 and found that siRNA stably inhibited the expression of FAM222A-AS1 in two CRC cell lines (**Figure 2B**). Among the siRNAs, siRNA-1 had the highest knockdown efficiency. We then investigated the effect of FAM222A-AS1 on CRC cell proliferation. A CCK-8 assay demonstrated that knockdown of FAM222A-AS1 slowed the growth of CRC cells (**Figure 2C**). Colony formation assays showed that colony numbers were significantly reduced after FAM222A-AS1 silencing (**Figure 2D**). Transwell assays were performed to detect the effects of FAM222A-AS1 on the migration and invasion of CRC cells. Our results showed that the migration and invasion abilities of CRC cells were significantly decreased by FAM222A-AS1 downregulation (**Figure 2E**). Taken together, these results indicate that FAM222A-AS1 knockdown repressed CRC cell proliferation, invasion, and migration.

To investigate the role of FAM222A-AS1 in the growth of CRC *in vivo*, stable HCT116 and SW620 cell models transfected with Dox-inducible shFAM222A-AS1 were constructed. To identify the knockdown effect and the cell function of the stable cell model, we performed qRT-PCR to assess the expression of CRC cells before and after Dox induction. Our results demonstrated that Dox-induced efficiency was more than 95% (**Figure S3**), and the FAM222A-AS1 expression in the stable cell model after 2.5 µg/ml Dox induction was lower than that in the non-Dox-induced group (**Figure 2F**). The CCK-8 assays and colony assays (**Figures 2G, H**) showed that FAM222A-AS1 knockdown inhibited CRC cell proliferation, while transwell assays demonstrated that the migration and invasion abilities decreased in the FAM222A-AS1 knockdown group (**Figure 2I**).

To confirm the effect of FAM222A-AS1 upregulation on CRC growth *in vitro*, we constructed a stable cell model transfected with the Dox-induced pINDUCER20 plasmid containing the FAM222A-AS1 sequence. After Dox induction, FAM222A-AS1 expression was significantly higher, approximately 200-fold (**Figure 2J**), in CRC cells than in the control group (-Dox). The colony assays showed that the colony numbers and size were larger for FAM222A-AS1 overexpression (+Dox) than in the control group (-Dox) (**Figure 2K**). The scratch assays demonstrated that FAM222A-AS1 overexpression (+Dox) promoted CRC cell migration (**Figure 2L**).

### FAM222A-AS1 Promote CRC Tumor Growth *In Vivo*

**Figure 3:**
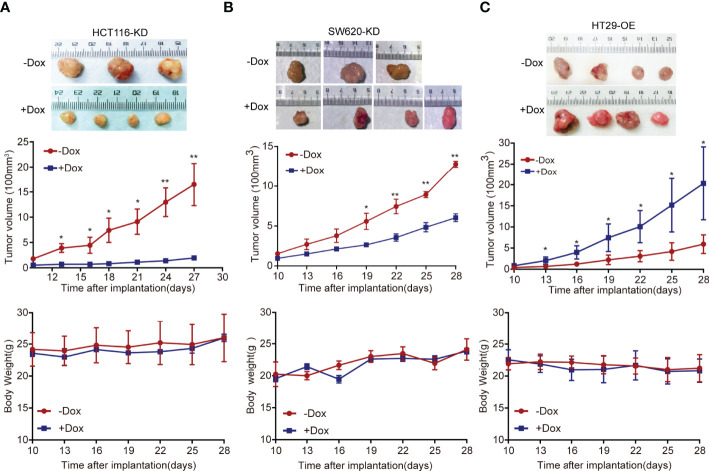
The FAM222A-AS1 knockdown inhibits tumour growth *in vivo*, and overexpression of FAM222A-AS1 promote the tumour growth in mice. **(A)** HCT116 cells were stably transfected with the FAM222A-AS1-shRNA vector and injected subcutaneously into nude mice. Compared with the control group (-Dox), FAM222A-AS1 downregulation (+Dox) inhibited tumor growth. **(B)** SW620 cells were stably transfected with the FAM222A-AS1-shRNA vector and injected subcutaneously into nude mice. Compared with the control group (-Dox), FAM222A-AS1 downregulation (+Dox) inhibited tumor growth. **(C)** HT29 cells were stably transfected with the FAM222A-AS1-overexpression vector and injected subcutaneously into nude mice. Compared with the control group (-Dox), FAM222A-AS1 overexpression (+Dox) promote tumor growth. HT29-OE, HT29 stable cells transinfected with FAM222A-AS1 overexpression vectors. (*P < 0.05, **P < 0.01).

The stable cell models HCT116 and SW620 were injected into nude mice to establish a xenograft tumor model. In the xenograft tumor model, the Dox-induced group tumors (+Dox) derived from cells transfected with shFAM222A-AS1 were smaller than those without Dox induction (-Dox). Knockdown of FAM222A-AS1 significantly suppressed tumor volume. The body weights of nude mice in the two groups did not differ significantly (**Figures 3A, B**). This study demonstrated that FAM222A-AS1 may play an important role in promoting CRC growth *in vivo*.

Furthermore, a subcutaneous xenograft model was constructed to validate the biological function of FAM222A-AS1 overexpression *in vivo*. Consistent with the *in vitro* results, FAM222A-AS1 overexpression (+Dox) significantly increased tumor volume compared to that in the control group (-Dox). The body weights of mice in the two groups were not significantly different (**Figure 3C**). Our findings indicated that FAM222A-AS1 upregulation promoted CRC cell proliferation both *in vitro* and *in vivo*.

### FAM222A-AS1 May Target MYH9 by Functioning as a miR-Let-7f Sponge

**Figure 4:**
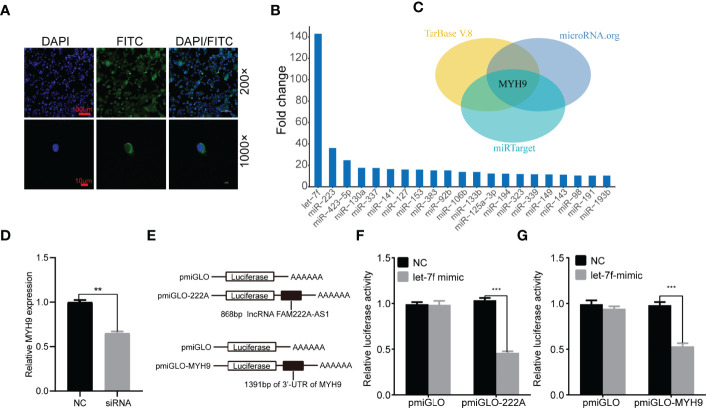
FAM222A-AS1 may directly interacted with miR-let-7f and MYH9 is a target of miR-let-7f. **(A)** The location of FAM222A-AS1 (green) in HCT116 cells was determined by FISH assay. DAPI-stained nuclei are blue. **(B)** qRT-PCR of multiplex miRNA data of FAM222A-AS1 probe affinity purification, in HCT116 cells. Data shown are normalized values for the enriched miRNAs (>10 fold). **(C)** The MYH9 was the target of miR-let-7f based on three miRNA target prediction websites (TarBase, microRNAorg and miRTarget). **(D)** MYH9 was downregulated when the FAM222A-AS1 was silenced by siRNA. **(E)** Schematic representation of pmiGLO firefly luciferase reporter construction. 868 bp FAM222A-AS1 sequence and 1391 bp of MYH9 3’-UTR sequence were cloned and constructed in pmiGLO firefly luciferase reporter. **(F, G)** Luciferase activity analysis of HCT116 cells co-transfected with pmirGLO dual luciferase miRNA target expression vector, together with negative control and miR-let-7f mimics. "(**P < 0.01 and ***P < 0.001).

To elucidate the molecular mechanism underlying FAM222A-AS1, we performed FISH and bioinformatic analysis to detect the subcellular localization of FAM222A-AS1. As shown in **Figure 4A**, we found that FAM222A-AS1 was predominantly located in the cytoplasm, indicating that FAM222A-AS1 may act as a ceRNA to capture miRNAs, leading to the release of specific miRNA-targeted transcripts. To investigate this hypothesis, we carried out RNA pull-down assays and found that miR-let-7f had the highest abundance among 21 potential binding miRNAs (**Figure 4B** and **Table 1**). In addition, we constructed FAM222A-AS1 luciferase reporters to detect the role of miR-let-7f in the regulation of FAM222A-AS1 activity. After FAM222A-AS1 was knockdown by transfecting the siRNA of FAM222A-AS1, the expression of MYH9 was assessed by qRT-PCT. We found that MYH9 was downregulated when the FAM222A-AS1 was silenced (**Figure 4D**). After transfection, we observed that overexpression of miR-let-7f significantly inhibited FAM222A-AS1 luciferase reporter activity (**Figures 4E, F**). These experiments revealed that FAM222A-AS1 may function as a sponge for miR-let-7f.

**Table 1:** The results of RNA-pulldown for miRNA assay in HCT116 cells using FAM222A-AS1 probes (FAM222A-AS1/control >10).

miRNA	FAM222A-AS1/control
miR-let-7f	143.01
miR-223	36.25
miR-423-5p	24.76
miR-130a	17.63
miR-337	17.51
miR-141	16.45
miR-127	16.11
miR-153	16.00
miR-383	15.24
miR-92b	15.24
miR-106b	13.93
miR-133b	13.83
miR-125a-3p	12.38
miR-194	12.21
miR-323	11.88
miR-339	11.63
miR-149	11.47
miR-143	11.31
miR-98	10.41
miR-191	10.34
miR-193b	10.34

miRNA, microRNA.

Based on the interaction between FAM222A-AS1 and miR-let-7f, we explored the potential roles of miR-let-7f in CRC. A previous study indicated that miR-let-7f acts as a tumor suppressor in the progression of CRC, inhibiting the proliferation, migration, and invasion of CRC cells. Furthermore, miRNAs can regulate the expression of their target mRNAs by binding to the 3’-UTR of mRNA. We predicted the target genes of miR-let-7f *via* three miRNA prediction websites (TarBase, microRNAorg, and miRTarget) (**Figure 4C**), and found that MYH9, which was previously shown to be involved in the promotion of CRC cancer cell migration or invasion, was one of the target genes of miR-let-7f. To confirm that MYH9 is a possible target of miR-let-7f, a sequence of the 3’-UTR of MYH9 containing the miR-let-7f-binding sequence was employed to synthesize a luciferase reporter plasmid. The results of luciferase reporter analysis showed that co-transfection of a miR-let-7f mimic and an MYH9 plasmid strongly decreased luciferase activity (**Figure 4G**).

### FAM222A-AS1 May Regulate the AKT1/2 and GSK3α/β Signaling Pathways

To explore which signaling pathway FAM222A-AS1 can regulate, we screened human protein kinase phosphorylation variation with the overexpression of FAM222A-AS1. We found that the phosphorylation of AKT1/2 (S473) and GSK3α/β (S21/S9) was upregulated, along with the overexpression of FAM222A-AS1 in CRC cells (**Figure S4A**), which indicates that FAM222A-AS1 may regulate CRC cell proliferation and invasion *via* downstream AKT- and GSK3α/β-associated pathways. The results of western blotting verified that phosphorylation of AKT1/2 (S473) was indeed altered along with the expression of FAM222A-AS1 (**Figure S4B**).

## Discussion

Increasing evidence demonstrates that lncRNAs play key roles in the pathogenesis and progression of human cancers, including the prognosis of cancer patients. lncRNAs can regulate the biological functions of tumors by integrating them with other cellular miRNAs or proteins (24–26). In this study, we identified that FAM222A-AS1, FAM83H-AS1, and FEZF1-AS1 were upregulated in CRC tissues. Furthermore, patients with higher FAM222A-AS1, FAM83H-AS1, and FEZF1-AS1 had poor overall survival and disease-free survival compared to those with lower levels. Functional studies revealed that FAM222A-AS1 promoted growth and progression of CRC cells *in vitro* and *in vivo* by sponging miR-let-7f to promote the expression of MYH9, indicating a tumor-accelerant role in CRC. Although several CRC prognosis-associated dysregulated lncRNAs have also been identified, various studies have elucidated their function.

The biological functions of lncRNAs are based in no small part on their different subcellular localizations. Accumulating evidence has shown that lncRNAs located in the cytoplasm can regulate gene expression at the post-transcriptional level, such as acting as a ceRNA to protect the target gene from repression (27). Using bioinformatics analysis and RNA FISH assays, we found that FAM222A-AS1 was mainly localized in the cytoplasm, indicating its potential for functioning as a miRNA sponge. Subsequently, the RNA pull-down for miRNA indicated that FAM222A-AS1 was highly associated with miR-let-7f, which was further validated by a luciferase reporter assay. Together, our results revealed that FAM222A-AS1 could act as a ceRNA by sponging miR-let-7f in CRC.

MiR-let-7f involved in various physiological and pathological processes, including neural stem cell differentiation (28), angiogenesis (29), immunocyte regulation (30) and carcinogenesis (31). Let-7f is downregulated in various cancers and is associated with poor overall survival. Low plasma miR-let-7f levels in patients with non-small cell lung cancer (NSCLC) and ovarian cancer are associated with poor outcomes (32, 33). In addition, a study revealed that miR-let-7f-5p was simultaneously downregulated in plasma and stool samples from early-stage colorectal cancer (34). Our study indicated that FAM222A-AS1 can act as a ceRNA by sponging miR-let-7f to regulate the function of miR-let-7f in CRC.

MYH9 is closely related to the progression and poor prognosis of gastric and esophageal cancers, suggesting its potential role in promoting cancer. A previous study indicated that MYH9 has a key role in tumor cell invasion (35, 36). Previous studies have shown that MYH9 overexpression correlates with clinicopathological parameters and poor prognosis in gastric cancer (37) and epithelial ovarian cancer (38). LncRNA HULC knockdown repressed gastric cancer progression, at least partly by regulating the miR-9-5p/MYH9 axis (39). MYH9 was also highly expressed in most colorectal cancer patients, and was significantly associated with patient age, clinical stage, lymph node metastasis, and metastasis distance (40). The positive rates of MYH9 protein in colorectal adenocarcinoma tissues and para-cancerous tissues were 51.6% and 11.5%, respectively. Furthermore, a proteogenomic analysis of CRC also revealed that high MYH9 expression was associated with shorter overall survival and disease-free survival (41). Qing Liao and Park et al. provided evidence that MYH9 may serve as a novel CRC metastasis-associated protein (40, 42). In short, MYH9 plays an important role in CRC metastasis. A previous study illustrated that miR-let-7f can suppress the invasion and metastasis of gastric cancer by directly binding to the 3’UTR of MYH9 (43). However, the association of miR-let-7f and MYH9 in CRC has not been examined. Our results, which showed that the FAM222A-AS1/miR-let-7f/MYH9 axis promotes CRC cell proliferation and invasion, provided the first evidence that the tumor metastasis-associated gene MYH9 is a target of miR-let-7f in CRC, and that FAM222A-AS1 may be a novel prognosis-associated lncRNA and therapeutic candidate for CRC. An in-depth study of the association between FAM222A-AS1, miR-let-7f, and MYH9, and the detailed mechanism will be investigated in our future studies.

A recent study reported that MYH9 promotes CRC cell growth and metastasis *via* activation of MAPK/AKT signaling in colorectal cancer (44). Our human phosphorylation protein screening assay indicated that FAM222A-AS1 was significantly associated with the variations in AKT1/2 (S473) and GSK3α/β phosphorylation, implying that FAM222A-AS1 may regulate the progression of CRC *via* AKT1/2 and GSK3α/β-associated signaling pathways, which needs to be verified in a future study.

There are several limitations in our study. First, the lack of important clinical-pathological features of clinical patients in our study, such as tumor invasion depth, distant metastasis, histological type, and TNM stage, influenced the results of the correlation between the expression of FAM22A-AS1 and the prognosis of CRC patients. Second, the sample size of clinical tumor tissues was too small, limiting the further analysis of clinical data. Third, there were several mice with varying degrees of scratch during the experiment, resulting a different number of tumor samples in mice between the two groups. Fourth, we only explored the possible potential mechanism of FAM222A-AS1 promoting the progression of CRC from multiple dimensions. However, more studies are needed to confirm the specific regulatory mechanisms.

## Conclusions

In conclusion, we identified three prognosis-related lncRNAs, FAM222A-AS1, FAM83H-AS1, FEZF1-AS1 as oncogenes in CRC, and upregulation of these lncRNAs was associated with poor prognosis. High expression of FAM222A-AS1 promoted tumor growth *in vivo* and CRC cell proliferation, migration, and invasion. FAM222A-AS1 may act as a sponge for miR-let-7f to attenuate its repressive effect on MYH9. Our results provide a better understanding of the role of lncRNAs in CRC progression and a potential therapeutic target and prognostic predictor of this malignancy. Further study needs to be done to reveal the specific molecular mechanism.

##  Data Availability Statement

The original contributions presented in the study are included in the article/**Supplementary Material**. Further inquiries can be directed to the corresponding authors.

## Ethics Statement

The animal study was reviewed and approved by Changhai Hospital, Second Military Medical University.

## Author Contributions

HS, WC, and MM designed the study. HS, WC, CS, and MS analyzed the data and revised the manuscript. MS wrote the manuscript. MS and YL performed most of the experiments. JZ, ZC, LY, and FZ performed some of the experiments. All of the authors discussed the results, reviewed and approved the final manuscript.

## Funding

This work was financially supported by National Key Research and Development Program to HS (No. 2017YFC1309200) and the National Natural Science Foundation of China (NSFC, 81672888).

## Conflict of Interest

The authors declare that the research was conducted in the absence of any commercial or financial relationships that could be construed as a potential conflict of interest.

## Publisher’s Note

All claims expressed in this article are solely those of the authors and do not necessarily represent those of their affiliated organizations, or those of the publisher, the editors and the reviewers. Any product that may be evaluated in this article, or claim that may be made by its manufacturer, is not guaranteed or endorsed by the publisher.
