# Supplementary material for: The Long Non-Coding RNA FAM222A-AS1 Negatively Modulates MiR-Let-7f to Promote Colorectal Cancer Progression
Source: Front Oncol. 2022 May 12;12:764621. doi: 10.3389/fonc.2022.764621 (PMC9133450; doi:10.3389/fonc.2022.764621)
Supplement: Supplementary file 1 [file DataSheet_1.docx]

Supplementary Material


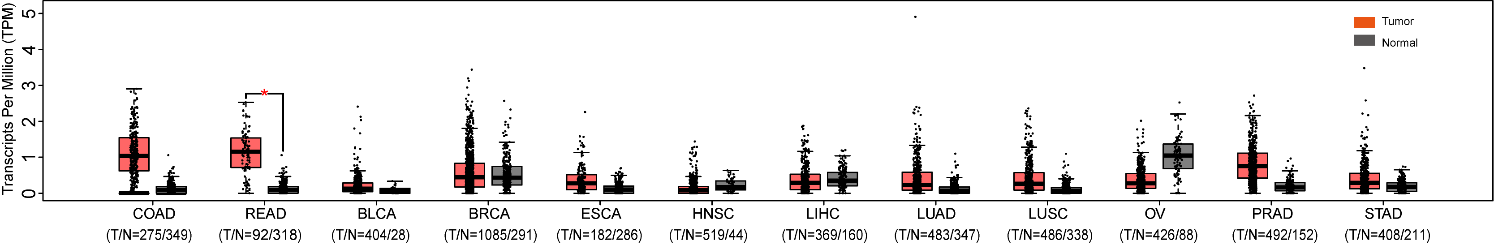


**Supplementary Figure1** Relative expression of FAM222A-ASS1 in tumor tissues and corresponding nontumor tissues of different cancer types provided by GEPIA (<http://gepia.cancer-pku.cn>). COAD, Colon adenocarcinoma; READ, Rectum adenocarcinoma; BLCA, Bladder urothelial carcinoma; BRCA, Breast invasive carcinoma; ESCA, Esophageal carcinoma; HNSC, Head and neck squamous cell carcinoma; LIHC, Liver hepatocellular carcinoma; LUAD, Lung adenocarcinoma; LUSC, Lung squamous cell carcinoma; OV, Ovarian serous cystadnocarcinoma; PRAD, Prostate adenocarcinoma; STAD, Stomach adenocarcinoma


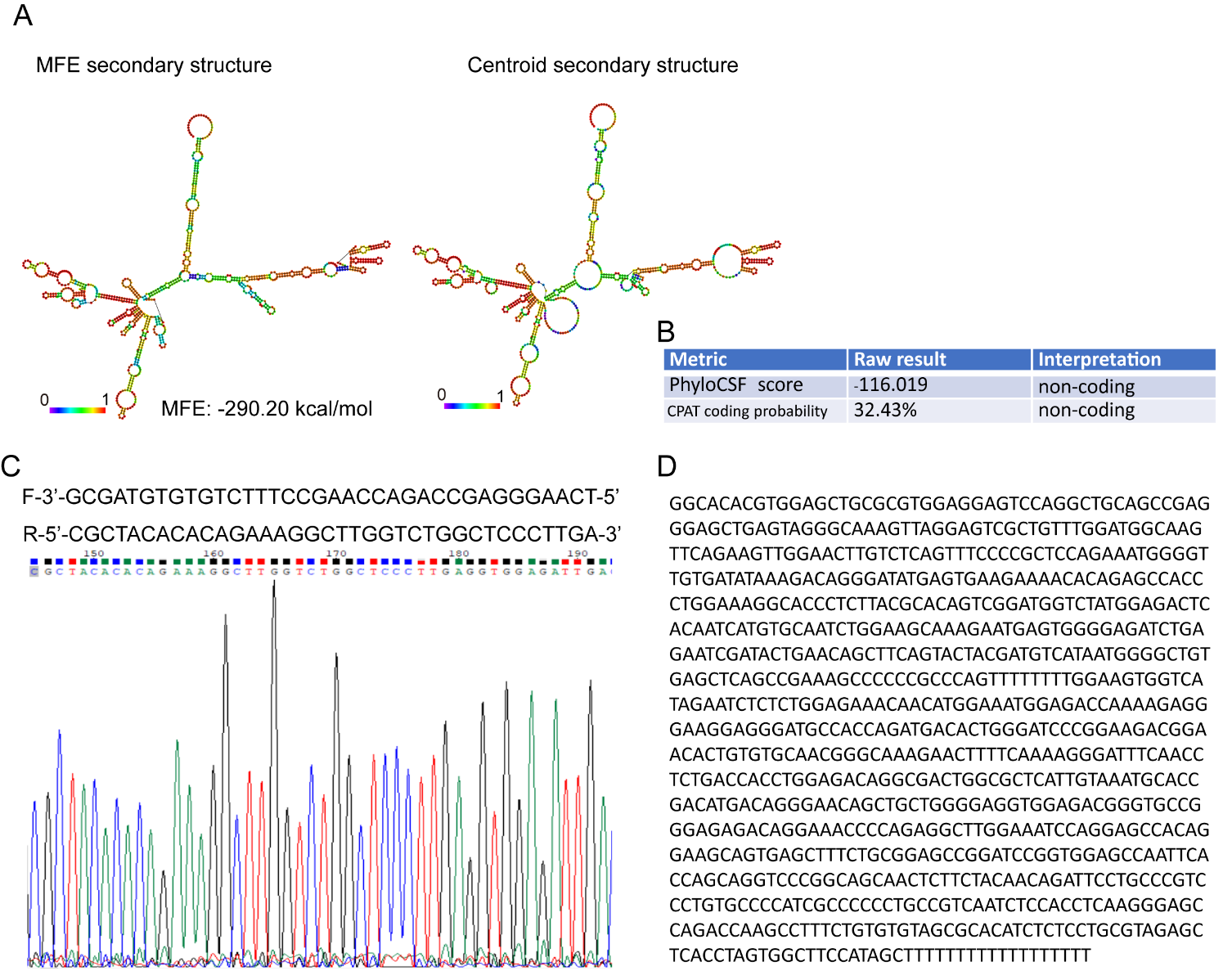


**Supplementary Figure2.** Characterization of full-length human FAM222A-AS1 in CRC cells and protein coding potential as well as secondary structure of FAM222A-AS1. (a) The RNA secondary structure of FAM222A-AS1 predicted via RNAfold Webserver (http://rna.tbi.univie.ac.at/) based on minimum free energy (MFE) and partition function. Color scale from weak to strong in line with the changes of color from purple to red indicates the predictive confidence. (b) Protein coding potential of FAM222A-AS1 predicted via LNCipedia (https://lncipedia.org). (c-d) The gene sequencing of partial synthesis full length of FAM222A-AS1 (c) and the nucleotide sequence of full-length human FAM222A-AS1 (d).


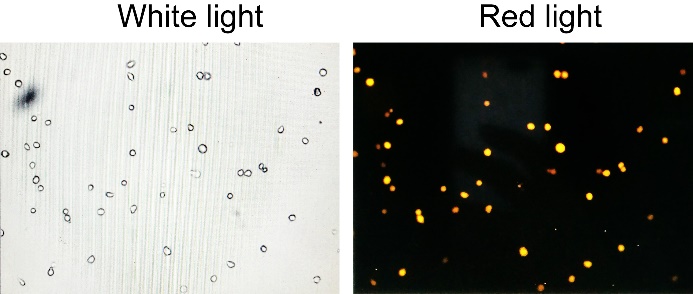


**Supplementary Figure3.** The Dox-induce efficiency of HCT116 cells. Dox, doxycycline; the red light means the cells whose shRNA was induced expression by doxycycline.


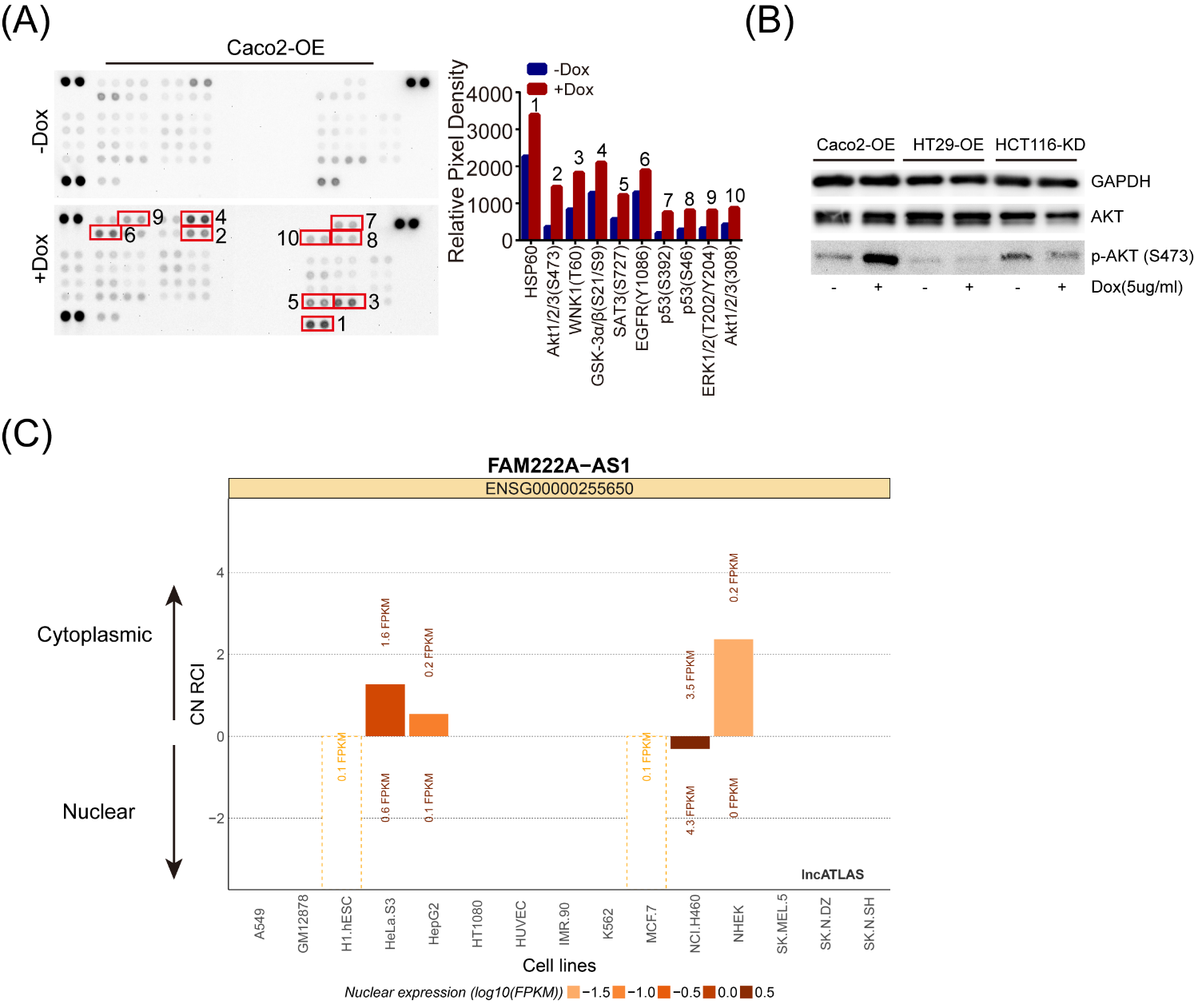


**Supplementary Figure4.** The screening of FAM222A-AS1-associated phosphorylation protein via human phospho-kinase array. (a) The results of human phospho-kinase array between control and FAM222A-AS1 overexpression group. (b) The validation of the AKT(S473) phosphorylation after the FAM222A-AS1 knockdown and overexpression by western blot. (c) The cellular localization of FAM222A-AS1 based on the bioinformation analysis (lncATLAS).


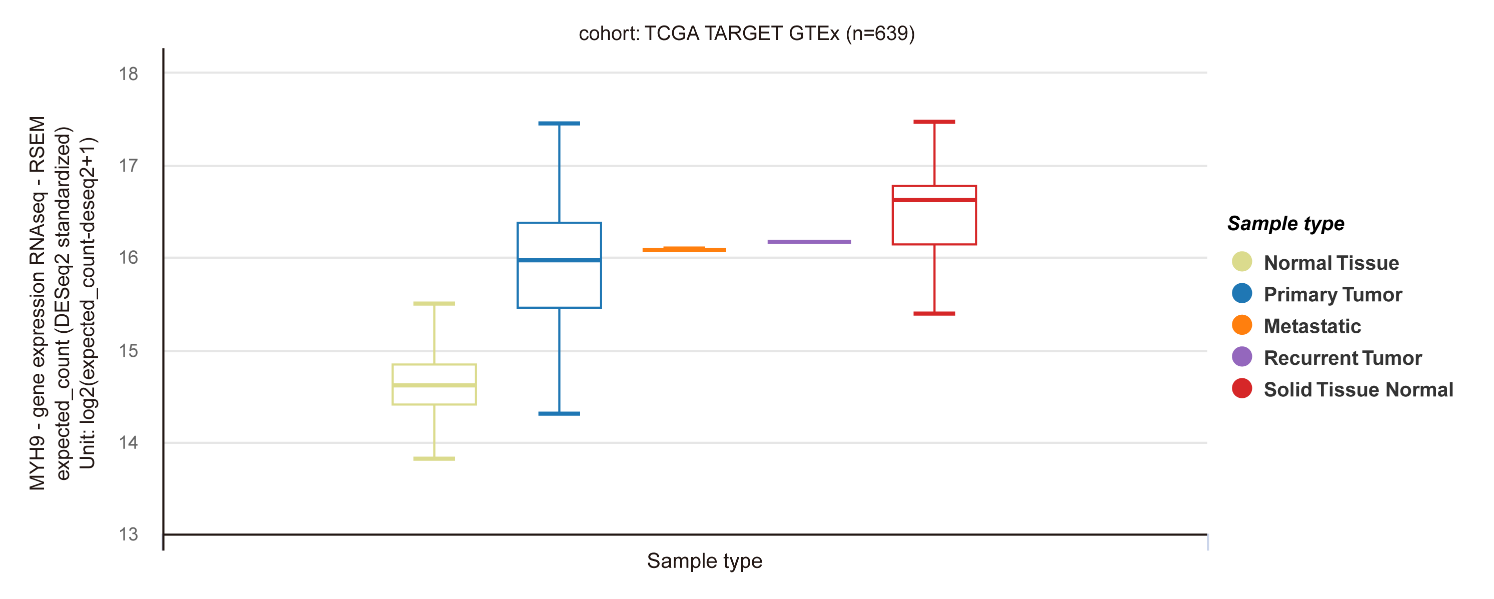


**Supplementary Figure5.** The expression of MYH9 in different colorectal sample tissues based on the TCGA database.

**Supplementary Table1.** The qRT-PCR primers of four CRC prognosis-associated dysregulated lncRNAs and shRNA of FAM222A-AS1.

| Names | Primer (5’→3’) |
| --- | --- |
| FAM222A-AS1-F | GAAAGGCACCCTCTTACGCACAGTC |
| FAM222A-AS1-R | CATCCCTCCTTCCCTCTTTTGGTCTC |
| FAM83H-AS1-F | GTACCTTGCCCTTGGTGGAATCTT |
| FAM83H-AS1-R | AACAGCAAACATCATCCTCAATGGT |
| FEZF1-AS1-F | AGAGGCTATGACTCAGGGTT |
| FEZF1-AS1-R | TGTTGCTCCACAGTAAAGGT |
| LINC00265-F | CCAGGGCGGAAGGAAGACGAG |
| LINC00265-R | GAACCTGCTTACAGAACATCACGGG |
| Actin-F | CAGCAAGCAGGAGTATGACG |
| Actin-R | GAAAGGGTGTAACGCAACTAA |
| 5'miR30PCR-xhoI-F | cagaaggctcgagaaggtatattgctgttgacagtgagcg |
| 3'miR30PCR-EcoRI-F | ctaaagtagccccttgaattccgaggcagtaggca |

**Supplementary Table2.** The screening of the CRC-prognosis dysregulated lncRNAs based on bioinformation analysis (The Cancer LncRNome Atlas).

| lncRNA | Ensembl_ID | Parametric p-value | FDR | Normal | Tumor | Fold-change T/N |
| --- | --- | --- | --- | --- | --- | --- |
| HAND2-AS1 | ENSG00000237125 | 0.004918 | 0.0154 | 3.31 | 0.053 | 0.016013 |
| LINC01082 | ENSG00000269186 | < 1e-07 | < 1e-07 | 7.08 | 0.73 | 0.102987 |
| FENDRR | ENSG00000268388 | < 1e-07 | < 1e-07 | 13.53 | 2.12 | 0.156986 |
| ZNF667-AS1 | ENSG00000166770 | < 1e-07 | < 1e-07 | 3.72 | 0.81 | 0.216920 |
| SERTAD4-AS1 | ENSG00000203706 | < 1e-07 | < 1e-07 | 2.6 | 0.68 | 0.260417 |
| GNG12-AS1 | ENSG00000232284 | < 1e-07 | < 1e-07 | 0.32 | 0.099 | 0.311526 |
| HHIP-AS1 | ENSG00000248890 | 4e-07 | 3.80e-06 | 0.81 | 0.27 | 0.335570 |
| LINC00959 | ENSG00000237489 | < 1e-07 | < 1e-07 | 0.25 | 0.1 | 0.403226 |
| BOLA3-AS1 | ENSG00000225439 | 3.51e-05 | 0.000214 | 0.58 | 0.24 | 0.420168 |
| LINC00886 | ENSG00000240875 | 0.003287 | 0.011 | 0.41 | 0.18 | 0.448430 |
| KCNJ2-AS1 | ENSG00000267365 | 3.3e-06 | 2.58e-05 | 0.73 | 0.33 | 0.456621 |
| PCAT6 | ENSG00000228288 | 4e-07 | 3.80e-06 | 0.71 | 1.49 | 2.083333 |
| LINC00265 | ENSG00000188185 | < 1e-07 | < 1e-07 | 0.63 | 1.5 | 2.380952 |
| SNHG12 | ENSG00000197989 | < 1e-07 | < 1e-07 | 3.63 | 9.02 | 2.500000 |
| CASC9 | ENSG00000249395 | 0.000422 | 0.00184 | 0.96 | 2.69 | 2.777778 |
| FAM83H-AS1 | ENSG00000203499 | < 1e-07 | < 1e-07 | 0.92 | 6.92 | 7.692308 |
| FAM222A-AS1 | ENSG00000255650 | < 1e-07 | < 1e-07 | 0.058 | 0.45 | 7.692308 |
| FEZF1-AS1 | ENSG00000230316 | 0.000014 | 9.34e-05 | 0.012 | 0.65 | 52.63158 |

**Supplementary Table3.** The univariate Cox analysis of CRC prognosis-associated dysregulated lncRNAs.

| LncRNAs | Down/Up regulation | High n (%) | Low n (%) | HR (95%CI) | Log rank *P*-value |
| --- | --- | --- | --- | --- | --- |
| HAND2-AS1 | Down | 129(74.1) | 45(25.9) | 0.369(0.188-0.723) | 0.004 |
| LINC01082 | Down | 134(77.0) | 40(23.0) | 0.400(0.200-0.800) | 0.010 |
| FENDRR | Down | 124(71.3) | 50(28.7) | 0.381(0.196-0.739) | 0.004 |
| ZNF667-AS1 | Down | 136(78.2) | 38(21.8) | 0.411(0.204-0.828) | 0.013 |
| SERTAD4-AS1 | Down | 59(33.9) | 115(66.1) | 0.325(0.134-0.786) | 0.013 |
| HHIP-AS1 | Down | 91(52.3) | 83(47.7) | 0.277(0.133-0.579) | 0.001 |
| LINC00959 | Down | 106(60.9) | 68(39.1) | 0.455(0.233-0.890) | 0.022 |
| BOLA3-AS1 | Down | 140(80.5) | 34(19.5) | 0.461(0.222-0.955) | 0.037 |
| LINC00886 | Down | 79(45.4) | 95(54.6) | 2.368(1.182-4.744) | 0.015 |
| KCNJ2-AS1 | Down | 130(74.7) | 44(25.3) | 0.457(0.299-0.910) | 0.026 |
| PCAT6 | Up | 111(63.8) | 63(36.2) | 2.085(1.192-7.983) | 0.020 |
| LINC00265 | Up | 95(54.6) | 79(45.4) | 2.507(1.189-5.286) | 0.016 |
| SNHG12 | Up | 102(58.6) | 72(41.4) | 0.418(0.2122-0.823) | 0.012 |
| CASC9 | Up | 79(45.4) | 95(54.6) | 2.128(1.082-4.186) | 0.029 |
| FAM83H-AS1 | Up | 74(42.5) | 100(57.5) | 2.099(1.075-4.100) | 0.030 |
| FAM222A-AS1 | Up | 39(22.4) | 135(77.6) | 2.349(1.064-5.183) | 0.034 |
| FEZF1-AS1 | Up | 99(56.9) | 75(43.1) | 3.342(1.486-7.514) | 0.004 |

**Supplementary Table4**. Baseline information of 50 colorectal cancer patients.

|  | All (n=50) | FAM222A-AS1 | |  |
| --- | --- | --- | --- | --- |
| Variables | n (%) | Low | High | *P* |
| Age (mean ± SD) | 59.5±11.8 | 57.7±13.1 | 60.6±11.1 | 0.424 |
| Sex, n (%) |  |  |  |  |
| Male | 28 (56.0) | 9 (32.1) | 19 (67.9) | 0.335 |
| Female | 22 (44.0) | 10 (45.5) | 12 (54.5) |  |
| Location, n (%) |  |  |  |  |
| Colon | 26 (52.0) | 10 (38.5) | 16 (61.5) | 0.944 |
| Rectum | 24 (48.0) | 9 (37.5) | 15 (62.5) |  |
| Family history, n (%) |  |  |  |  |
| Yes | 6 (12.0) | 3 (50.0) | 3 (50.0) | 0.519 |
| No | 44 (88.0) | 16 (36.4) | 28 (63.6) |  |
| CEA, n (%) |  |  |  |  |
| <5ng/ml | 29 (58.0) | 11 (37.9) | 18 (62.1) | 0.772 |
| >5ng/ml | 19 (38.0) | 8 (42.1) | 11 (57.9) |  |
| Missing | 2 (4.0) |  |  |  |
| CA19-9, n (%) |  |  |  |  |
| <37U/mL | 41 (82.0) | 15 (36.6) | 26 (63.4) | 0.289 |
| >37U/mL | 2 (4.0) | 0 (0) | 2 (100) |  |
| Missing | 7 (144.0) |  |  |  |
| Tumor size (mean ± SD) | 26.1±39.1 | 32.8±51.0 | 22.0±29.8 | 0.350 |
| Lymphatic metastasis, n (%) |  |  |  |  |
| Yes | 19 (38.0) | 8 (42.1) | 11 (57.9) | 0.640 |
| No | 31 (62.0) | 11 (35.5) | 20 (64.5) |  |
| B-raf, n (%) |  |  |  |  |
| WT | 41 (82.0) | 14 (34.1) | 27 (65.9) | 0.314 |
| MT | 2 (4.0) | 0 (0) | 2 (100) |  |
| Missing | 7 (14.0) |  |  |  |
| K-ras, n (%) |  |  |  |  |
| WT | 20 (40.0) | 4 (20.0) | 16 (80.0) | 0.101 |
| MT | 23 (46.0) | 10 (43.5) | 13 (56.5) |  |
| Missing | 7 (14.0) |  |  |  |
| Chemotherapy |  |  |  |  |
| Yes | 40 (80.0) | 15 (37.5) | 25 (62.5) | 0.884 |
| No | 10 (20.0) | 4 (40.0) | 6 (60.0) |  |
| Status |  |  |  |  |
| Death | 49 (98.0) | 18 (36.7) | 31 (63.3) |  |
| Alive | 1 (2.0) | 1 (100) | 0 (0) |  |

* Fisher's exact test; # t’ test; SD: standard deviation; Chemotherapy: postoperative chemotherapy; the missing data were not involved in the analysis

WT: wild type; MT: mutant type

**RNA sequence:**

**MYH9 3UTR**

GCCTCTTCTCCTGCAGCCTGAGATGGATGGACAGACAGACACCACAGCCTCCCCTTCCCAGACCCCGCAGCACGCCTCTCCCCACCTTCTTGGGACTGCTGTGAACATGCCTCCTCCTGCCCTCCGCCCCGTCCCCCCATCCCGTTTCCCTCCAGGTGTTGTTGAGGGCATTTGGCTTCCTCTGCTGCATCCCCTTCCAGCTCCCTCCCCTGCTCAGAATCTGATACCAAAGAGACAGGGCCCGGGCCCAGGCAGAGAGCGACCAGCAGGCTCCTCAGCCCTCTCTTGCCAAAAAGCACAAGATGTTGAGGCGAGCAGGGCAGGCCCCCGGGGAGGGGCCAGAGTTTTCTATGAATCTATTTTTCTTCAGACTGAGGCCTTTTGGTAGTCGGAGCCCCCGCAGTCGTCAGCCTCCCTGACGTCTGCCACCAGCGCCCCCACTCCTCCTCCTTTCTTTGCTGTTTGCAATCACACGTGGTGACCTCACACACCTCTGCCCCTTGGGCCTCCCACTCCCATGGCTCTGGGCGGTCCAGAAGGAGCAGGCCCTGGGCCTCCACCTCTGTGCAGGGCACAGAAGGCTGGGGTGGGGGGAGGAGTGGATTCCTCCCCACCCTGTCCCAGGCAGCGCCACTGTCCGCTGTCTCCCTCCTGATTCTAAAATGTCTCAAGTGCAATGCCCCCTCCCCTCCTTTACCGAGGACAGCCTGCCTCTGCCACAGCAAGGCTGTCGGGGTCAAGCTGGAAAGGCCAGCAGCCTTCCAGTGGCTTCTCCCAACACTCTTGGGGACCAAATATATTTAATGGTTAAGGGACTTGTCCCAAGTCTGACAGCCAGAGCGTTAGAGGGGCCAGCGGCCCTCCCAGGCGATCTTGTGTCTACTCTAGGACTGGGCCCGAGGGTGGTTTACCTGCACCGTTGACTCAGTATAGTTTAAAAATCTGCCACCTGCACAGGTATTTTTGAAAGCAAAATAAGGTTTTCTTTTTTCCCCTTTCTTGTAATAAATGATAAAATTCCGAGTCTTTCTCACTGCCTTTGTTTAGAAGAGAGTAGCTCGTCCTCACTGGTCTACACTGGTTGCCGAATTTACTTGTATTCCTAACTGTTTTGTATATGCTGCATTGAGACTTACGGCAAGAAGGCATTTTTTTTTTTTAAAGGAAACAAACTCTCAAATCATGAAGTGATATAAAAGCTGCATATGCCTACAAAGCTCTGAATTCAGGTCCCAGTTGCTGTCACAAAGGAGTGAGTGAAACTCCCACCCTACCCCCTTTTTTATATAATAAAAGTGCCTTAGCATGTGTTGCAGCTGTCACCACTACAGTAAGCTGGTTTACAGATGTTTTCCACTGAGCATCACAATAAAGAGAACCATGTGCTACGA

**FAM222A-AS1**

GGCACACGTGGAGCTGCGCGTGGAGGAGTCCAGGCTGCAGCCGAGGGAGCTGAGTAGGGCAAAGTTAGGAGTCGCTGTTTGGATGGCAAGTTCAGAAGTTGGAACTTGTCTCAGTTTCCCCGCTCCAGAAATGGGGTTGTGATATAAAGACAGGGATATGAGTGAAGAAAACACAGAGCCACCCTGGAAAGGCACCCTCTTACGCACAGTCGGATGGTCTATGGAGACTCACAATCATGTGCAATCTGGAAGCAAAGAATGAGTGGGGAGATCTGAGAATCGATACTGAACAGCTTCAGTACTACGATGTCATAATGGGGCTGTGAGCTCAGCCGAAAGCCCCCCGCCCAGTTTTTTTTGGAAGTGGTCATAGAATCTCTCTGGAGAAACAACATGGAAATGGAGACCAAAAGAGGGAAGGAGGGATGCCACCAGATGACACTGGGATCCCGGAAGACGGAACACTGTGTGCAACGGGCAAAGAACTTTTCAAAAGGGATTTCAACCTCTGACCACCTGGAGACAGGCGACTGGCGCTCATTGTAAATGCACCGACATGACAGGGAACAGCTGCTGGGGAGGTGGAGACGGGTGCCGGGAGAGACAGGAAACCCCAGAGGCTTGGAAATCCAGGAGCCACAGGAAGCAGTGAGCTTTCTGCGGAGCCGGATCCGGTGGAGCCAATTCACCAGCAGGTCCCGGCAGCAACTCTTCTACAACAGATTCCTGCCCGTCCCTGTGCCCCATCGCCCCCCTGCCGTCAATCTCCACCTCAAGGGAGCCAGACCAAGCCTTTCTGTGTGTAGCGCACATCTCTCCTGCGTAGAGCTCACCTAGTGGCTTCCATAGC
